# Editorial: Management of *Fusarium* Species and their Mycotoxins in Cereal Food and Feed

**DOI:** 10.3389/fmicb.2017.01543

**Published:** 2017-08-17

**Authors:** Thomas Miedaner, Daniela Gwiazdowska, Agnieszka Waśkiewicz

**Affiliations:** ^1^State Plant Breeding Institute, University of Hohenheim (720) Stuttgart, Germany; ^2^Department of Natural Science and Quality Assurance, Faculty of Commodity Science, Poznań University of Economics Poznań, Poland; ^3^Department of Chemistry, Faculty of Wood Technology, Poznań University of Life Sciences Poznań, Poland

**Keywords:** Fusarium, mycotoxins, cereals, food contamination, feeding

*Fusarium* species are pathogenic fungi that appear in all wheat and maize growing areas worldwide. They are only lowly specialized and one *Fusarium* species can infect several hosts and host organs. The worldwide most recognized species, *Fusarium graminearum*, for example, readily infects wheat, triticale, barley, oat, rye as well as maize and the infected organs are similarly diverse: seedling, roots, stems/stalks, ears (Becher et al., 2013). The most prevalent diseases are Fusarium head blight (FHB) in wheat and barley and Fusarium ear rot (FER) in maize caused by cereal infecting *Fusarium* pathogens.

Infection of cereals by *Fusarium* spp. reduces grain yield in the first line. A yield loss of 1 Mg ha^−1^ was predicted to occur at 19% FHB incidence (Salgado et al., 2015). Additionally, grain size and baking quality are affected, and the harvested grain is contaminated with mycotoxins, especially A and B trichothecenes, fumonisins, and the estrogenic zearalenone that are harmful for humans and livestock (Gallo et al., 2015; Stoycho, 2015). Among these mycotoxins deoxynivalenol (DON) and its acetylated forms 3-ADON and 15-ADON, are considered to be the most important, but a whole spectrum of other mycotoxins differing in chemical structure and toxicity may appear (Stoycho, 2015), because several *Fusarium* species can naturally co-occur in the same host tissue. Due to their heat stability, *Fusarium* mycotoxins occur in the whole cereal supply chain from the farmer to the customer providing problems to all stakeholders. In the European Union and many other countries, maximum levels for DON, T-2 and HT-2 toxins, zearalenone, and fumonisins in human food and guidance levels in animal feeding are implemented (Ferrigo et al., 2016). Consequently, grain contaminated with DON above 1,250 μg kg^−1^, the threshold level allowed in food established by the EU, may be rejected or priced down by grain buyers. It is, therefore, of utmost importance to prevent the formation of mycotoxins or at least to reduce their concentration. Cumulative direct production and price impacts between 1998 and 2000 due to FHB on wheat and barley from the northern Great Plains and the Central States of the USA were estimated at $871 million over the period, with additional secondary economic losses of $1.8 billion (Nganje et al., 2004).

The main drivers of the cereal supply chain concerning *Fusarium* diseases are the seed and chemical industry, the farmers, and the storage and processing companies. In the field, weather, crop species, cultivars, *Fusarium* species/isolates and management practices are the main components affecting disease severity and mycotoxin contamination. To control *Fusarium* diseases and mycotoxins all these stakeholders should work together (Wegulo et al., 2015) as illustrated in this Research Topic.

At the beginning of the cereal supply chain stands the seed industry with the aim to improve host resistance. Growing resistant cultivars minimizes *Fusarium* incidence, severity and mycotoxin concentrations in an environmentally friendly and cost-effective way. This affords to detect resistance sources, to bring them into actual breeding material and to develop commercially successful cultivars. One strategy to achieve this aim is to search in native sources for FHB resistance and to identify the responsible genes (QTL) in a segregating population (McCartney et al.). This is a time-consuming procedure because FHB resistance is based on an array of genes with small effects that work together additively and are affected by environment. Consequently, the use of molecular markers should accelerate the introgression. An additional challenge in FHB resistance breeding is combining resistance with superior agronomic and quality characteristics (Clark et al.). Their results indicate that rare segregants within native wheat populations can be found that combine the most important traits when the populations are large enough. Although a lot of effort has been put into breeding resistant varieties in the last two decades, we still have no idea on the function of these resistance genes. Lahlali et al. showed that the lignification pathway and callose deposition can play a role in confining the fungus to the inoculation site. This might lead to candidates of biochemical markers for selecting FHB resistance in the lab. Another route for reaching the same goal is discussed by the review of Atanasova-Penichon et al. on the contribution of grain antioxidant secondary metabolites to the mechanisms of plant resistance to *Fusarium* and mycotoxin accumulation.

Another strategy for controlling FHB is the use of fungicides that have to be sprayed during flowering of wheat to be effective. Finding new fungicides by the chemical industry is a time-consuming and extremely costly procedure. Frac et al. developed a microplate assay to efficiently test substances for their fungicidal activity toward several *Fusarium* isolates in a more efficient way. Also biological antagonists or molecules could play a role in this framework as reviewed by Alberts et al.

The main stage of *Fusarium* development and potential mycotoxin hazard are the commercial fields (Ferrigo et al., 2016). Here, we face the pathologist's square with climate and weather, *Fusarium* as pathogen, the crop as host and farmers' management practices as the major drivers. FHB infections need high humidity during flowering while the temperature is normally not a limiting factor. The results of Scala et al. clearly show that the environmental conditions at field level and soil management practices may drive FHB outbreak and mycotoxin contamination even in a growing area suitable for cropping durum wheat like in Southern Italy. Similarly, in Norwegian oats rainy periods are necessary for *Fusarium* dispersal. Additionally, soil and straw management by the farmer are important factors (Wegulo et al., 2015). *Fusarium* infections are largely reduced when soil tillage is practiced before sowing wheat or as less straw as possible from the previous crop remains on the soil surface (Hofgaard et al.).

Hofgaard et al. has emphasized the importance of *Fusarium* species given the fact that in Europe we have mostly several species infecting the same crop, like in Norwegian oats, where *F. avenaceum, F. graminearum, F. culmorum*, and *F. langsethiae* have been isolated. A similar topic was followed in a fundamental study of Pasquali et al. where he and his 31 coauthors collected data on the occurrence of trichothecene genotypes of *F. graminearum* and *F. culmorum* in Europe. In *F. graminearum*, the predominant genotype was 15-acetyldeoxynivalenol (15-ADON) (82.9%), and in *F. culmorum* 3-ADON (59.9%). In the latter species, however, the nivalenol (NIV) genotype accounted for the remaining 40.1%. These data are available from a freely accessible and updated database (http://www.catalogueeu.luxmcc.lu). A totally different occurrence of chemotypes was found in Brazilian wheat where isolates from the *Fusarium graminearum* species complex were classified as NIV (55%), 15-ADON (43%), and 3-ADON (2%) chemotypes by PCR (Tralamazza et al.) illustrating that these chemotype analyses have to be performed in each region. To understand the evolution and role of trichothecene chemotypes a comparative gene expression study of nine out of 16 genes was accomplished (Amarasinghe and Fernando). One important outcome was that relative expression of *TRI* genes was higher in 3-ADON producing strains compared to 15-ADON and NIV strains. Analyzing the transcriptomes of *F. graminearum* cells infecting living, actively defending wheat heads *vs*. dead wheat tissue showed that in the living plant much higher toxin production is promoted (Boedi et al.).

The interaction of the four factors in the field complex results in the actual DON level of the harvest. Rainy weather, aggressive *Fusarium* isolates, susceptible crops and suboptimal management by the farmer can lead to a DON contamination surpassing legislative limits. Foroud et al. provided a deeper insight into the role of DON in disrupting protein synthesis of the host plant. This might provide in future possibilities to develop trichothecene remediation strategies. Such strategies could be important steps for reducing mycotoxin concentrations in grain storage centers or food processing industries. They include the use of bacterial biodegradation pathways that are capable of transforming DON to a non-toxic stereoisomer (He et al.). Perczak et al. and Kalagatur et al. found significant effects of selected essential oils extracted from diverse plants on degradation of zearalenone *in vitro* and on downregulating the expression of genes involved in zearalenone production in maize, respectively. In an excellent review Vanhoutte et al. summarize several approaches to reduce mycotoxins by chemical removal, physical binding, or microbial degradation. At the end, they provide the features of an ideal biodegrading and detoxifying agent that has still to be detected.

An even broader focus on this topic is given by Alberts et al. who review pre-harvest and post-harvest strategies for controlling fumonisin-producing Fusaria and their toxins in maize, including essential oils and microbial biodegradation but also resistance breeding and genetic engineering. This review stretches over the whole cereal supply chain and brings us back to the main aim of *Fusarium* research: to enhance food and feed safety by avoiding *Fusarium* infection and/or decreasing their effects. We have today accumulated much knowledge and a large number of strategies that include breeding resistant varieties, developing fungicides or antagonists, best management practices of the farmers, and possibilities of mycotoxin binding or degradation in storage and processing facilities (McMullen et al., 2012). The main challenge that remains is to combine the most effective measures in an integrative approach for combating *Fusarium* species wherever they occur.

## Author contributions

All authors listed have made a substantial, direct and intellectual contribution to the work, and approved it for publication.

### Conflict of interest statement

The authors declare that the research was conducted in the absence of any commercial or financial relationships that could be construed as a potential conflict of interest.
